# Change in Anthropometric Parameters of the Posture of Students of Physiotherapy after Three Years of Professional Training

**DOI:** 10.1155/2014/719837

**Published:** 2014-01-23

**Authors:** Joanna Glista, Teresa Pop, Aneta Weres, Ewelina Czenczek-Lewandowska, Justyna Podgórska-Bednarz, Justyna Rykała, Justyna Leszczak, Karolina Sowa, Wojciech Rusek

**Affiliations:** ^1^Institute of Physiotherapy of University of Rzeszow, Warszawska 26A Street, 35-205 Rzeszow, Poland; ^2^REHAMED-CENTER, Rehabilitation Center, Tajęcina 66a, 36-002 Tajęcina, Poland

## Abstract

*Introduction and Aim*. A physiotherapist's occupation requires high physical fitness and a properly functioning neuromuscular system. Working with patients is not always performed in accordance with the rules of work ergonomics. The aim of this paper was to verify the possible changes in the posture of students of physiotherapy after three years of professional training. *Material and Methods*. The sample group consisted of 30 randomly chosen students of physiotherapy. Each person was examined twice (at the age of 20 and at the age of 23). Both examinations were performed by the same researcher. The ultrasound system ZEBRIS Pointer was used for the examination; 17 parameters were analyzed in sagittal projection, frontal projection, and transversal projection. *Results*. Statistical analysis revealed positive correlations between the 10 parameters in examination 1 and examination 2 which means that a person with a relatively higher level of obliquity in examination number 1 also had slightly different values in an examination conducted three years later. *Conclusion*. Studying physiotherapy and physical work with patients resulted in a considerable worsening of the students' posture. It is advisable to educate students on ergonomics and the rules of safety and hygiene while working as a physiotherapist in order to protect the therapists' health.

## 1. Introduction

Changes in a man's posture may be caused by many factors, such as impaired muscle tone, presence of defect or impairment of organ of hearing or sight, presence of congenital defects, playing on asymmetrical music instruments, practicing asymmetrical sport disciplines (e.g., fencing), incorrect posture during daily activities, for example, during learning, watching TV, working on the computer, or doing professional work [1–4].

A physiotherapist's occupation requires high physical fitness and a properly functioning neuromuscular system. Working with patients is not always performed in accordance with the rules of work ergonomics. Lack of regulation of patient's bed and long-term work in a leaned or sitting position are merely the examples of many inconveniences that physiotherapists have to deal with during their daily work.

Despite the fact that critical periods in a man's posture shaping take place around the 7th year of life and the period of pubertal spurt, the posture is completely shaped during the period between 18 and 20 years of life [5, 6]. As a consequence students in the initial period of their education are also endangered with postural defects as a result of incorrect motor activity.

Lack of sufficient knowledge and experience in the ergonomics of the physiotherapist's work as well as the load connected with this occupation may cause a series of unfavourable changes in a young organism. The most common group of diseases are the so called work-related musculoskeletal disorders. They include popular backaches, omalgia, wrist, or thumb dysfunctions. They affect the majority of physiotherapists and other representatives of health care with various frequency and intensity [7–10].

An expedient and necessary action is to analyze and observe the development of body posture of the most vulnerable occupational groups, ranging from the earliest period of vocational education. The scientific substantiation beginning with the evolution of body posture will take the appropriate preventive and early action. This will also contribute to reducing the number of physical therapists with disorders of the musculoskeletal system.

## 2. Paper's Purpose

The aim of this paper is to verify the possible changes in the posture of students of physiotherapy after three years of professional training.

## 3. Material and Method

The sample group consisted of 30 randomly chosen students of physiotherapy at the University of Rzeszow, including 24 women (80%) and 6 men (20%). The random choice was organized by the main researcher who placed 60 small pieces of paper with consecutive numbers in a nontransparent container, which was in accordance with the number of students of the first year of physiotherapy. The research students whose numbers on the pieces of papers were the same as those on the students' list were qualified for the research.

The group of 30 people was chosen to reflect the characteristics of the population of the 1st year of physiotherapy at our university.

Inclusion criteria for the research:

- age 19-20,
- a student of the 1st year of physiotherapy,
- lack of established diseases of the musculoskeletal system,
- chosen in the process of random selection for the research group.

Each person was examined twice. In this study, the experimental pattern called the one group technique was used. The first examination was performed at the beginning of the first year of the first-cycle studies (at the age of 20) and the second one was performed at the end of the third year of the first-cycle studies (at the age of 23). In order to maintain the same test procedures, both examinations were performed by the same researcher in the same conditions with identical procedures and parameters of the device (Table 1).

During their three-year-long studies program, students had, among others, 1280 hours of practical classes and 45 hours of classes on physiotherapist's work ergonomics. Practical classes were conducted in the university study and on clinical wards directly with a patient. They included field subjects such as kinesitherapy, physiotherapy, massage, chiropractic and during student placement. During the course, 920 classes of professional student placement are realized. At the moment of research initiation the students did not signal any symptoms in the musculoskeletal system.

Examinations were conducted in the morning hours in the Anthropometry Centre of the Institute of Physiotherapy of the University of Rzeszow.

The ultrasound system ZEBRIS Pointer was used for examination. This device is a repeatable system used by many researchers for, among others, the analysis of body posture, spine mobility, and shoulder joint mobility [11–17].

**Figure 1:**
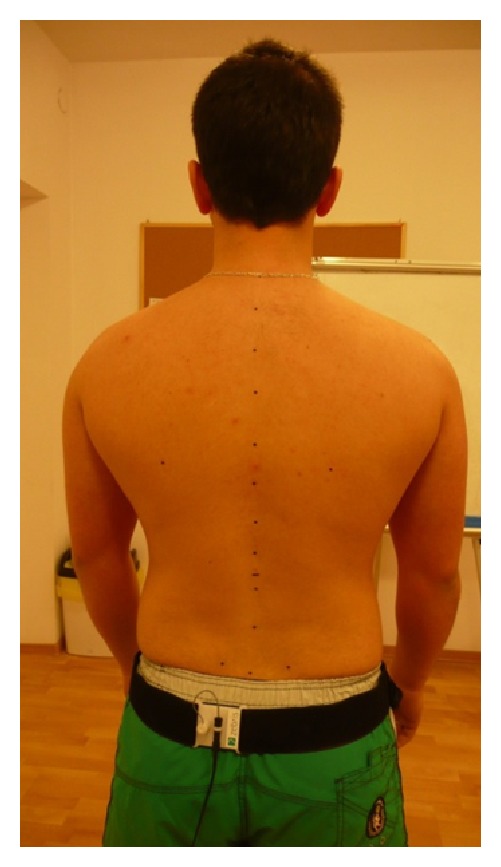
Examinee's position during the examination.

At the beginning of each examination, a belt with a sensor was placed on each examinee's waist and topographic points were marked on the skeleton of participants (Figure 1) as

- Acromion left and right,
- Spina iliaca posterior superior left and right,
- Spina iliaca anterior superior left and right,
- Crista iliaca left and right,
- point between thoracic and lumbar spine (Th12/L1),
- Angulus inferior scapulae left and right,
- line of the spinal column from C7 to sacral bone.

The examined person was turned back to the measuring unit without the shoes on, in a neutral position, with feet slightly apart, arms loosely on the sides, and looking straight on.

The distance between the examinee and the measuring unit was 80 cm. For the sake of preserving the examination conditions for future measurements in the place of examination, a line was placed indicating the patient's spot. The examination consisted in scanning the topographic points of the participant's skeleton by an ultrasound indicator. Points were scanned in the above mentioned order.

The result of the examination was at once recorded in a software and it was visible as a report with numeric data and charts presenting scanned points. The subject and the researcher did not have mobile phones during examination, so as not to falsify the results [11, 18].

Obtained research results were statistically analyzed. In order to compare the results of the examination, the values of descriptive statistics were presented for the arrangement of each of the parameters in consecutive examinations. The analysis of the significance of changes between examinations was conducted by means of Wilcoxon test. The results of this test (PW) were presented as descriptive statistics for differences between examinations. These results were completed by the value of Spearman's rank correlation coefficient, with which the dependence between results obtained in consecutive examinations was investigated. The selected test parameters are standard and programmed in the Zebris system.

The following parameters were analysed.

**Figure 2:**
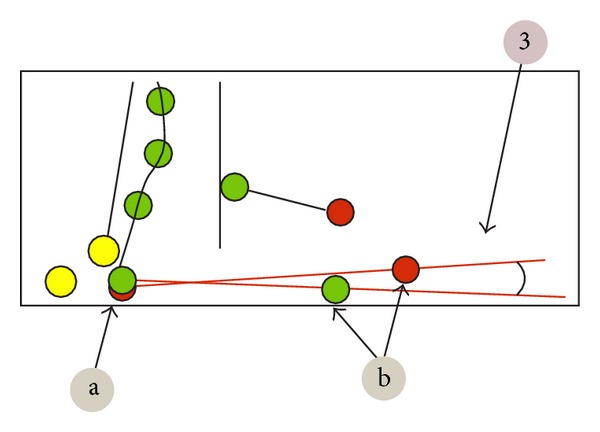
Analyzed parameters.

**Figure 3:**
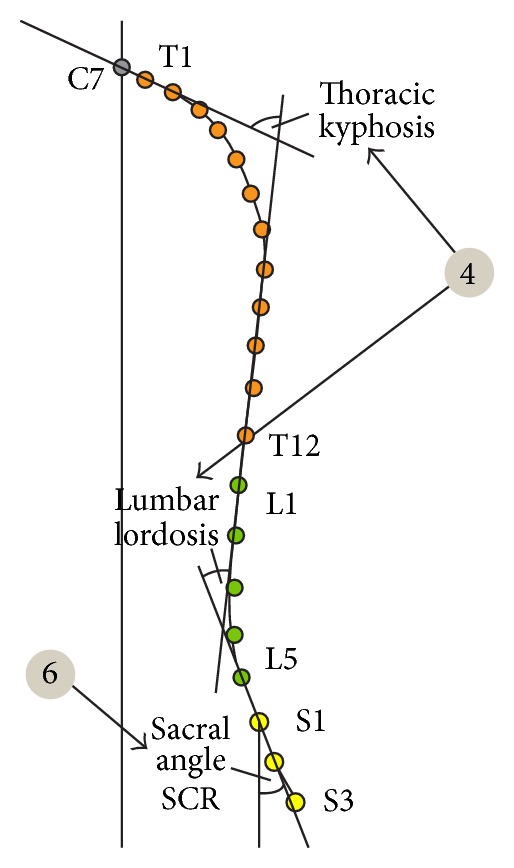
Analyzed parameters.

**Figure 4:**
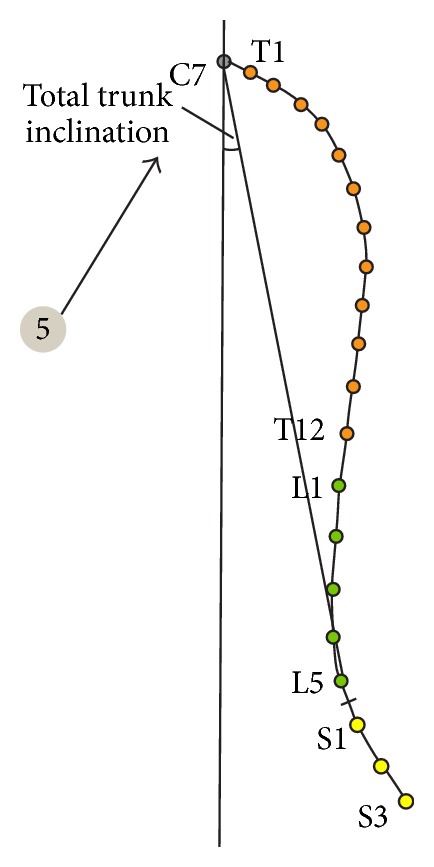
Analyzed parameters.

**Figure 5:**
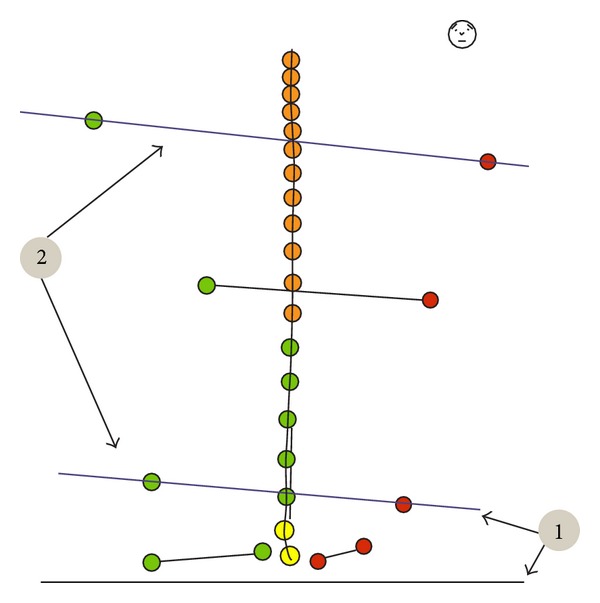
Analyzed parameters.

**Figure 6:**
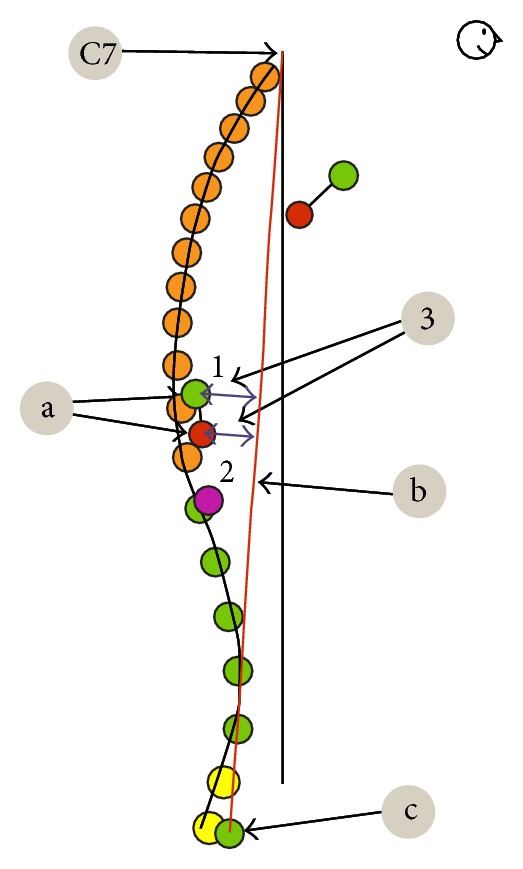
Analyzed parameters.

**Figure 7:**
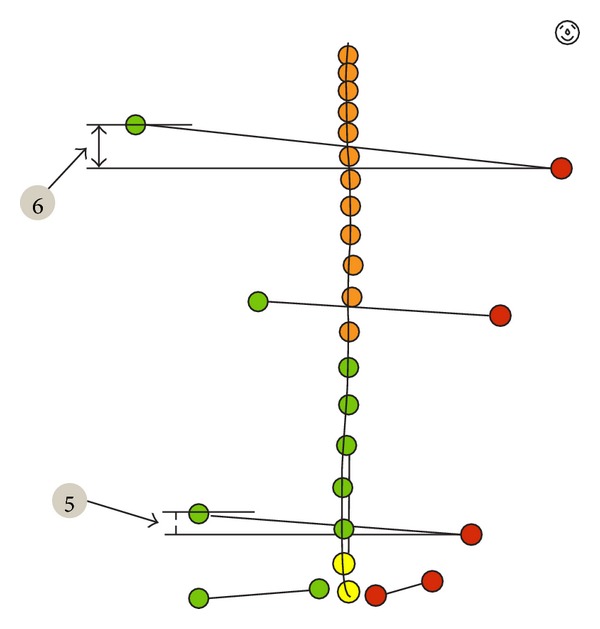
Analyzed parameters.

**Figure 8:**
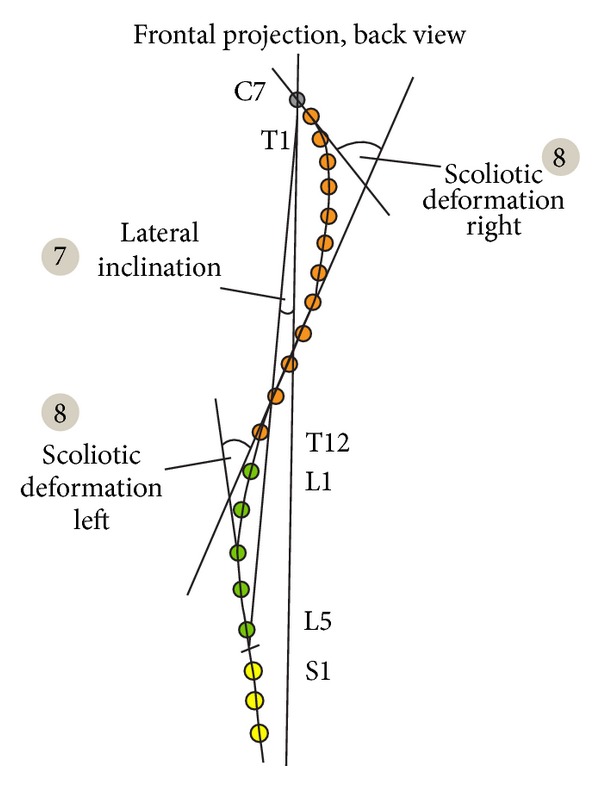
Analyzed parameters.

**Figure 9:**
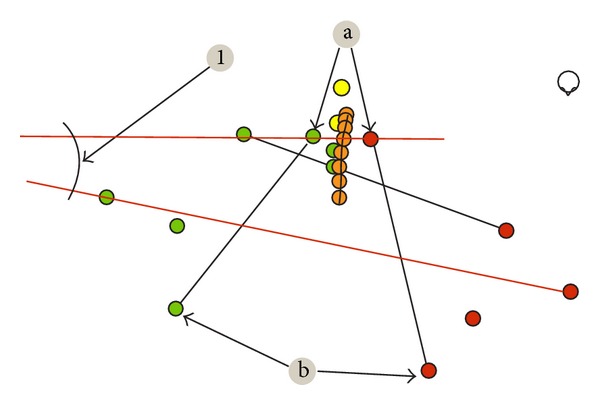
Analyzed parameters.

1. Sagittal projection.
  - Pelvic torsion—twist of the pelvis in degrees. It indicates the angle between the connecting line from Spina iliaca posterior superior right (a) to Spina iliaca anterior superior right (b) and the connecting line from Spina iliaca posterior superior left (a) to Spina iliaca anterior superior left (b) (Figure 2, point 3).
  - Thoracic kyphosis/lumbar lordosis—overall angle of the thoracic and the lumbar spine, respectively (Figure 3, point 4).
  - Sacral angle—the angle between the tangent in S1 and the frontal plane (Figure 3, point 6).
  - Total trunk inclination—the overall forwards inclination of the body is calculated using the angle of the connecting line from C7 to L5/S1 to the vertical axis (Figure 4, point 5).
2. Frontal projection.
  - Pelvic obliquity—lateral inclination of the pelvis in relation to the ground plane. It is represented by the angle between connecting line from Crista iliaca right to left and the ground plane (Figure 5, point 1).
  - Pelvic/shoulder obliquity—describes the obliquity of the shoulders (connecting line from Acromion left to right) to the pelvis (connecting line from Crista iliaca left to right) (Figure 5, point 2).
  - Scapula distance right/left—the maximum distance of the left (1) and right (2) scapula plane (a). The reference plane is the frontal plane (b). It is defined by the points C7 and both Spina iliaca superior posterior (Figure 6, points 1 and 2).
  - Scapula distance difference—the difference of the distance between left and right scapula and frontal plane (Figure 6, point 3).
  - Pelvic height difference—the height difference between Crista iliaca right and left. The point which is lower relative to the horizontal plane is the reference point (Figure 7, point 5).
  - Shoulder height difference—the height difference between Acromion right and left. The point which is lower relative to the horizontal plane is the reference point (Figure 7, point 6).
  - Lateral inclination—lateral deviation angle of the spinal crests. Angle between the sagittal plane and the connecting line from C7 to L5 (sum of the angle of each vertebra T1–L5) (Figure 8, point 7).
3. Transversal projection.
  - Pelvis/shoulder rotation—it indicates the angle of rotation between the connecting lines between both Spina iliaca posterior superior and between both shoulder points (Figure 9, point 1).

## 4. Results

**Figure 10:**
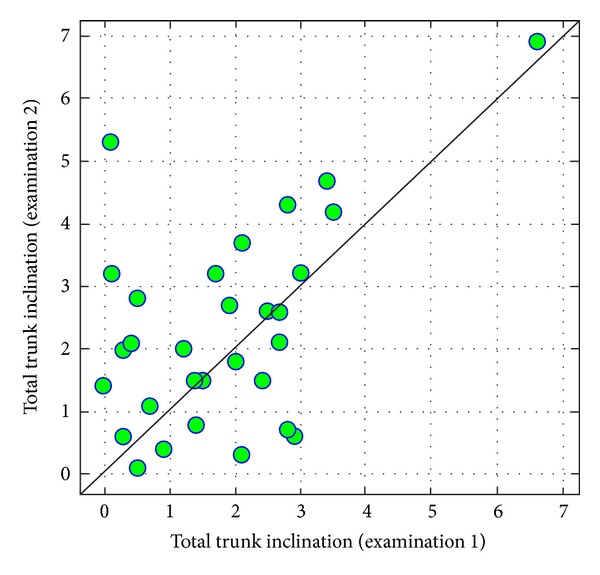
Comparison of total trunk inclination in examinations 1 and 2.

Statistical analysis of results of the first and second examination shows a certain increase in total trunk inclination. This effect is similar to the result considered to be statistically significant (value of test probability *P* < 0,05 is considered as such, in this case, *P* is slightly less than 0.10) (Figure 10).

**Figure 11:**
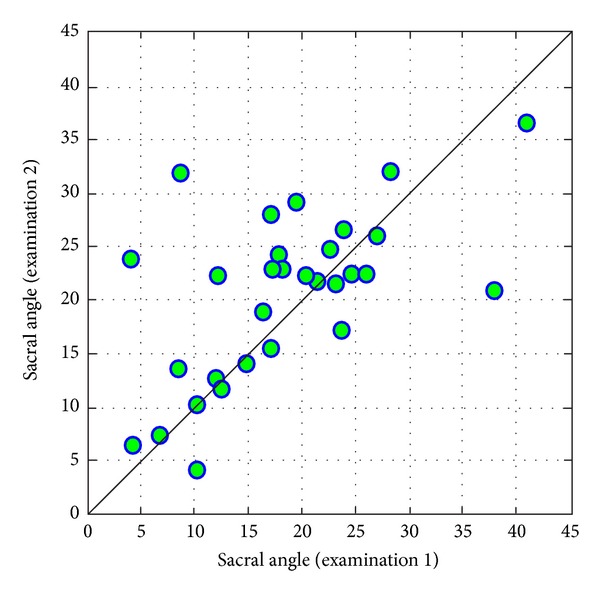
Comparison of sacral angle in examinations 1 and 2.

In the case of comparison of values of *sacral angle* in examinations 1 and 2, one can state a difference similar to the statistically significant one (*P* = 0,0999). In examination 2, on average, values of sacral angle are higher than in 1. (Figure 11).

There are no statistically significant changes or differences close to significance between the values of other evaluated parameters.

However, there were correlations indicated in case of parameters like thoracic kyphosis angle, lumbar lordosis angle, sacral angle, pelvic obliquity, pelvis/shoulders obliquity, distance between right spatula and coronal plane, distance between left spatula and coronal plane, difference in the height of the shoulder on the left side, and lateral inclination of the trunk, both right and left.

**Figure 12:**
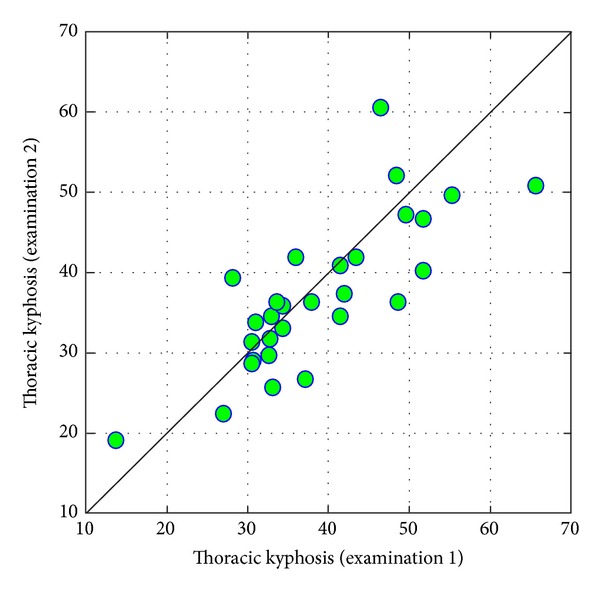
Comparison of values of thoracic kyphosis in examinations 1 and 2.

In case of *thoracic kyphosis*, it is worth emphasizing the high correlation between compared examinations, which means that people with a relatively higher value of thoracic kyphosis in the first examination also had higher results in the second examination (Figure 12).

**Figure 13:**
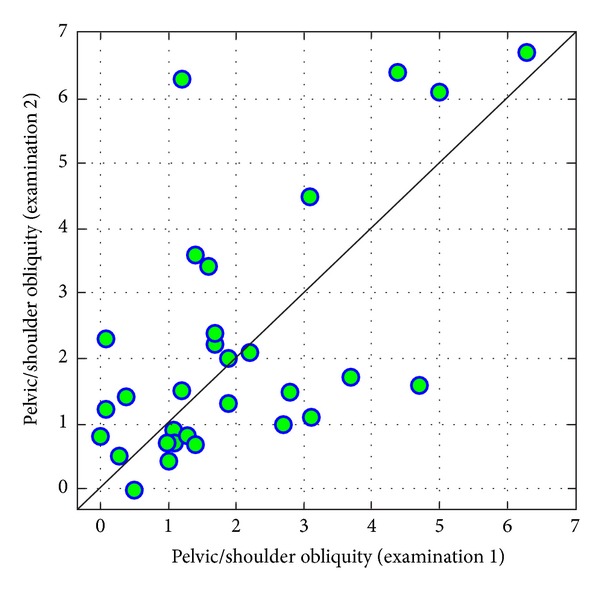
Comparison of values of pelvic/shoulder obliquity in examinations 1 and 2.

In case of *pelvic/shoulder obliquity*, parameter values in the compared measurements were correlated to some extent, which means that a person with a relatively higher level of obliquity in examination 1 also had slightly different values in an examination conducted three years later (Figure 13).

**Figure 14:**
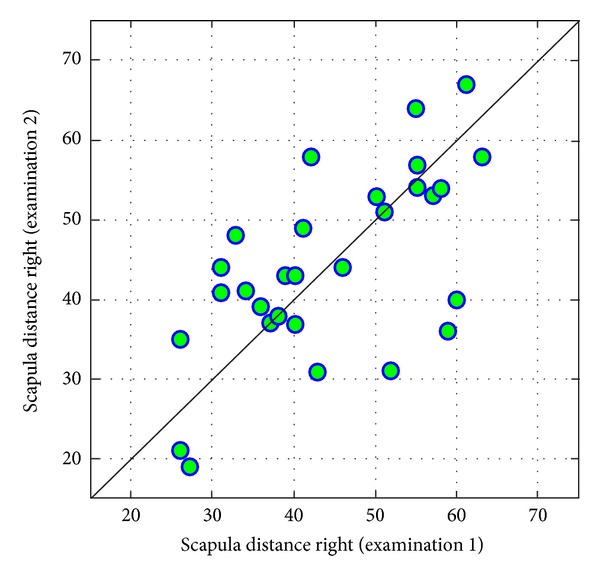
Comparison of values of right scapula distance from coronal plane in examinations 1 and 2.

**Figure 15:**
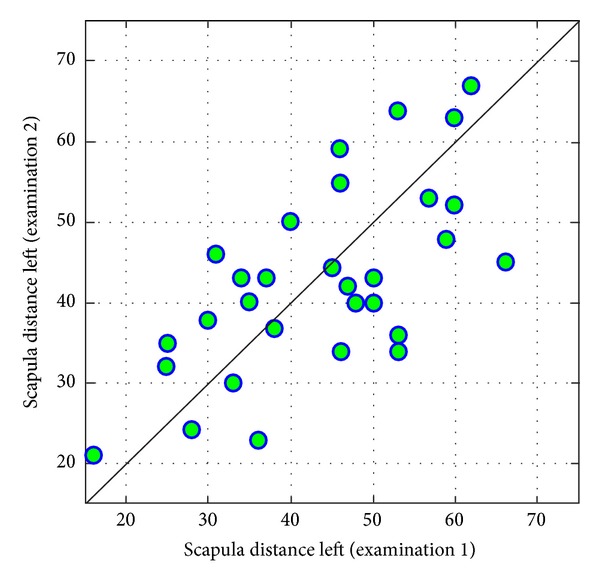
Comparison of values of left scapula distance from coronal plane in examinations 1 and 2.

Values of measurements of *scapula distance* conducted at three-year an intervals are correlated with each other on-average level (Figures 14 and 15).

**Figure 16:**
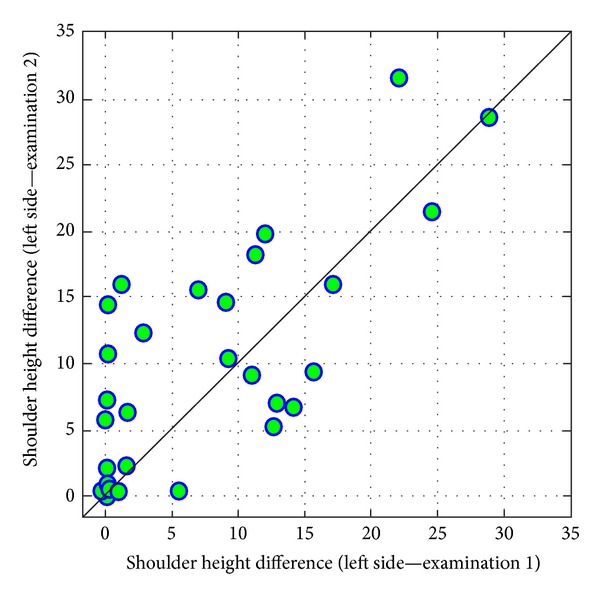
Comparison of values of shoulder height difference (left side) in examinations 1 and 2.

The more interesting result, in terms of a certain direction of changes between examinations, is connected with *shoulder height difference* (on the left side). There are mainly increases in values of the tested parameter. This difference can be considered similar to the statistically significant one (*P* = 0,0937) (Figure 16).

## 5. Discussion

Statistics indicate that more and more people complain about pain or other musculoskeletal disorders that they directly attribute to their professional work. Among the most common professions are office workers, professional drivers, workers of big factories, workers of furniture and car production plants, and employees of the medical sector, mostly physiotherapists [19–23].

Additionally, the analysis of the test results indicates that these disorders occur more often in the case of medical workers than in the case of industrial workers [24–26].

The physiotherapists constitute a professional group, that is, a group especially endangered with many negative health effects resulting from the performed work. It is connected with, among others, lifting weights, performing torsional movements, and maintaining an uncomfortable body position during the greater part of working time.

Students in the initial period of education seem to be especially exposed to such changes, as they are not provided with proper information on work ergonomics or this information on how work with a patient properly has not yet become a habit of the daily motor activity of the future physiotherapist.

As seen from the analysis conducted by Holder et al., the most common unfavourable situations during a physiotherapists' work were improper posture for a long working time (36%), weight lifting (35%), patient's carrying (30%), and conducting manual therapy (28%). Young people at aged between 21 and 30 years old, so beginning physiotherapists and students, reported the most problems [27]. The results of research of Alrowayeh et al. also indicate the fact that pains and inconveniences connected with the specifics of the physiotherapist's work were more often reported by young people aged between 20–40 years old. The authors state that such a result is probably connected with lack of experience, skills, elaborated technique, and knowledge of work ergonomics, and it is not connected with age and ageing of the physiotherapist's organism. What is more, they add that in the conducted research, almost half of the subjects reported at least 1 health problem connected with their profession. In comparison, in the USA it was about 60% of subjects and in Australia 91% of subjects [28, 29]. The present paper authors' own research also indicate that during their work physiotherapists assume incorrect body position which can lead to various kinds of pain and changes in body posture.

Adegoke et al. stated in their research that about 30% of the subjects suffered from ailments connected with musculoskeletal system disorders during physiotherapy studies. These disorders were the most frequently reported by young physiotherapists (up to 5 years from graduation—60%); however, this problem more often concerned students than people with 5–15-year-long professional experience. What is more, the subjects indicated the following factors as the most common ones predisposing them to such disorders: excessive number of patients during one day for one physiotherapist, long-term work in one position, lifting/carrying patients, performance of techniques of manual therapy, work in improper positions, and others [30].

As shown from our own research, small changes of posture occurred after 3 years in the case of students of physiotherapy. During that period, students attended classes according to the teaching program which included, among others, 1280 hours of practical classes. These classes consisted of theoretical and practical training in kinesitherapy, physiotherapy, massage, and manual therapy. What is more, within the framework of these classes, students attended clinical practice during which they broadened and strengthened acquired knowledge when working with a patient, and hence, already from the beginning of their studies they had contact with a patient for the entire 3 years of the first-cycle studies.

The conducted examinations prove that the total trunk inclination increased in the case of the students. It is probably the result of an improper position during work with a patient, by leaning. Such a position for the therapist may also predispose him/her to the development of back pain in the future, as young physiotherapists are very exposed to it. The analysis of results of research conducted in Australia by Nyland and Grimmer indicates that students of physiotherapy are in the high-risk group in terms of occurrence of pain syndromes in the lower spinal segment. Persons at the age of 20-21 years old are especially endangered [31].

Moreover, in the case of the majority of subjects there was a decrease in the value of the thoracic kyphosis angle. As it was evaluated on the basis of subjects' analysis and the graphic result of examination, in the first examination students had properly shaped physiological spine curvature. With regard to that, after 3 years they manifested a slight flattening of the thoracic kyphosis.

Another important thing is the fact that after 3 years, the values of left shoulder height and pelvic/shoulder obliquity parameters increased. It is yet another proof of the fact that during work with patients these persons focused on work, not thinking about their own health.

To sum up, it is worth refering to the work by Cromie et al. who created a guide with practical pieces of advice concerning maintenance of health and safety during work as a physiotherapist. The authors enumerate the rules that every therapist should comply with in order not only to improve, but also to remain fit. They also included rules created directly for students and candidates of physiotherapy stating that the candidates should be acquainted with the physical requirements necessary to perform this profession, choose a carrier path compliant with their physical skills, and take care of maintaining a proper level of personal motor skills. When it comes to students and young physiotherapists, they should not only care about the education of patients, but also of themselves in order to stay healthy and fit [32].

It is all commonly known, but is often forgotten or disregarded.

## 6. Conclusions

Studying physiotherapy as well as physical work with patients resulted in a considerable worsening of students' posture and resulted in the deterioration of the parameters: total trunk inclination, sacral angle, pelvic/shoulder obliquity, scapula distance, and shoulder height difference.

It is advisable to educate students on ergonomics and rules of safety and hygiene in their work as physiotherapists in order to protect therapists' health.

## Figures and Tables

**Table 1:** Comparison of differences of particular parameters in examination 1 and examination 2.

Examination 1 and examination 2	$\bar{x}$	Me	s	Min	Max	PW	R
PT (°)	0,5	0,5	2,9	−7,4	5,3	0,2494	0,11
TK (°)	−1,7	−1,6	6,4	−14,8	13,9	0,0999	0,81***
LL (°)	2,1	0,7	9,1	−17,0	25,7	0,4779	0,55**
TTI (°)	0,5	0,3	1,5	−2,3	5,2	0,0896	0,31
SA (°)	2,2	1,3	7,6	−17,1	23,2	0,0999	0,54**
PO (°)	0,3	0,5	1,2	−2,5	2,4	0,1567	0,40*
P/SO (°)	0,2	0,2	1,6	−3,1	5,1	0,4779	0,57***
SDR (mm)	1,0	1,0	11,1	−23,0	29,0	0,4935	0,48**
SDL (mm)	−0,9	−0,9	10,1	−21,0	15,0	0,7577	0,62***
SDD (mm)	1,0	1,0	6,6	−12,0	19,0	0,3991	0,04
PHDR (mm)	−0,1	0,0	4,8	−10,8	10,2	1,0000	0,36
PHDL (mm)	1,5	0,0	5,7	−10,1	13,0	0,2113	0,18
SHDR (mm)	0,5	0,0	4,7	−11,5	13,0	0,4703	0,23
SHDL (mm)	2,2	0,3	6,2	−7,6	14,7	0,0937	0,62***
LIR (°)	0,0	0,0	1,0	−2,5	3,0	0,9826	0,66***
LIL (°)	0,1	0,0	0,8	−1,7	2,7	0,2560	0,85***
P/S rotation (°)	−0,2	−0,8	2,8	−9,3	5,4	0,6733	0,12

PT: pelvic torsion; TK: thoracic kyphosis; LL: lumbar lordosis; TTI: total trunk inclination; SA: sacral angle; PO: pelvic obliquity; P/SO: pelvic/shoulder obliquity; SDR: scapula distance right; SDL: scapula distance left; SDD: scapula distance difference; PHDR/PHDL: pelvic height difference right/left; SHDR/SHDL: shoulder height difference right/left; LIR/LIL: lateral inclination right/left; P/S rotation: pelvic/shoulder rotation.
